# Zebrafish Caudal Fin Angiogenesis Assay—Advanced Quantitative Assessment Including 3-Way Correlative Microscopy

**DOI:** 10.1371/journal.pone.0149281

**Published:** 2016-03-07

**Authors:** Ruslan Hlushchuk, Daniel Brönnimann, Carlos Correa Shokiche, Laura Schaad, Ramona Triet, Anna Jazwinska, Stefan A. Tschanz, Valentin Djonov

**Affiliations:** 1 Institute of Anatomy, University of Bern, Bern, Switzerland; 2 Department of Biology, University of Fribourg, Fribourg, Switzerland; Medical College of Wisconsin, UNITED STATES

## Abstract

**Background:**

Researchers evaluating angiomodulating compounds as a part of scientific projects or pre-clinical studies are often confronted with limitations of applied animal models. The rough and insufficient early-stage compound assessment without reliable quantification of the vascular response counts, at least partially, to the low transition rate to clinics.

**Objective:**

To establish an advanced, rapid and cost-effective angiogenesis assay for the precise and sensitive assessment of angiomodulating compounds using zebrafish caudal fin regeneration. It should provide information regarding the angiogenic mechanisms involved and should include qualitative and quantitative data of drug effects in a non-biased and time-efficient way.

**Approach & Results:**

Basic vascular parameters (total regenerated area, vascular projection area, contour length, vessel area density) were extracted from *in vivo* fluorescence microscopy images using a stereological approach. Skeletonization of the vasculature by our custom-made software Skelios provided additional parameters including “graph energy” and “distance to farthest node”. The latter gave important insights into the complexity, connectivity and maturation status of the regenerating vascular network. The employment of a *reference point* (vascular parameters prior amputation) is unique for the model and crucial for a proper assessment. Additionally, the assay provides exceptional possibilities for correlative microscopy by combining *in vivo*-imaging and morphological investigation of the area of interest. The 3-way correlative microscopy links the dynamic changes *in vivo* with their structural substrate at the subcellular level.

**Conclusions:**

The improved zebrafish fin regeneration model with advanced quantitative analysis and optional 3-way correlative morphology is a promising *in vivo* angiogenesis assay, well-suitable for basic research and preclinical investigations.

## Introduction

Angiogenesis, the formation of new blood vessels from existing ones, is an extensively investigated process with an enormous medical, scientific, and economic impact. It plays a major role in cardiovascular disorders and cancer progression, which together account for about 2/3 of the worldwide mortality [1]. Accordingly, the number of registered clinical trials dealing with angiogenesis is impressive–around 4000 (http://clinicaltrials.gov). Prior to clinical trials, only 0.1% of the potential drug candidates pass the pre-clinical stage [2].

After tremendous investment of time, lab animals and resources, the sponsors and the investigating teams often end up being frustrated due to disappointing quantitative results of their studies. We believe that this situation could be improved through a better understanding of the effects of the drug candidate in an *in vivo*-system. The basic and pre-clinical research would greatly benefit from improved, powerful and simple assays [3]. The latter should help to evaluate the compound’s effects roughly but swiftly and cheap. According to the obtained results, a well-focused and precise “classical” pre-clinical study should be started. Similar situation is obvious in the field of angiogenic research, where the current animal models used are mainly of embryonic origin (e.g., zebrafish (0–5 dpf), chicken area vasculosa and chorioallantoic membrane) or lab rodents (see review by K. Norrby 2006). Some embryonic models are well accessible for *in vivo* observation, however, the blood vessels differ dramatically from the adult ones [4]. The adult rodent models, which are clinically more relevant, are expensive, experiments are rather time-consuming and technically demanding, especially in the mouse eye [5]. In addition, longer *in vivo*-observation and follow-up at reasonable resolution, as well as the quantification remain a challenge. The statement of Auerbach *et al* from 1991 is still actual: “Perhaps the most consistent limitation in all these studies and approaches has been the availability of simple, reliable, reproducible, quantitative assays of angiogenic response” [6]. In general, profound quantification of the angiogenic response is missing in most of the reported studies. In studies dealing with tumor angiogenesis, vascular density, as a number of blood vessels per field in so-called “hot-spots”, is estimated most of the time: the results obtained are biased, hardly reproducible and often controversial [7]. Moreover, the vascular density has no actual physiological meaning and provides no information about perfusion, maturation or vascular exchange surface (potential diffusion area) [8]. The lack of a profound quantification methodology and use of inappropriate embryonic models could, at least partially, contribute to the existing discrepancy between the positive effects in animal models and drug failure in clinical trials.

Teleost fish, including zebrafish, are able to regenerate heart, retina, spinal cord, and fins after a lesion [9]. The zebrafish has emerged as an alternative powerful model system to study human diseases, including a variety of neoplasms and hypoxia-related pathologies [10–13]. Despite more than 400 million years that separate the last common ancestor of zebrafish and humans, many zebrafish organs are remarkably similar to their human counterparts as at the anatomical, physiological, and molecular levels [14]. Ease of genetic manipulation is a prominent advantage of this vertebrate model system. Together with the lower space requirement and cost when compared to other vertebrate models [15], large-scale genetic screens are facilitated in zebrafish as a model organism [16;17]. The zebrafish caudal fin is mainly used to study different aspects of the regenerative process and this assay has gained popularity in the past decade [18]. The availability of the genetically modified zebrafish with green fluorescing endothelial cells made this model even more attractive for vital imaging of the post-injury angiogenesis. In 2006 this regeneration assay has been introduced as a non-embryonic (adult) angiogenesis model by Bayliss et al. [19]. In the mentioned study, Bayliss and coworkers also introduced a quantification of the vascularization of the regenerated fin by measuring the advances in vascularization and regeneration fronts. This quantitative analysis was not able to differentiate between various vascular patterns, but was a coarse and robust tool that could be used for distinguishing between total and partial inhibition of angiogenesis [19]. This new model of regenerative angiogenesis was to accelerate discovery of genes and drugs related to angiogenesis [20]. Azevedo and co-workers have recently shown that the caudal fin has an almost unlimited capacity to regenerate [21], making it another remarkable advantage of the model for research groups, including the ones in the field of angiogenesis.

While *in vivo*-assays are usually more time-consuming and difficult to perform than *in vitro*-assays [22], the unique structure of the zebrafish caudal fin makes it a suitable model in many ways. The distal tip of the fin is less than 150 μm thick and is organized in an almost single plane [23]. These features offer the possibility for repetitive *in vivo* observation at high resolution. This great advantage, combined with a stereological approach, allows profound quantification and overall description of the caudal fin vasculature.

In the present study, we have performed inhibition experiments using the PTK787 concentration range suggested by Bayliss et al. The great advantage of the suggested approach for analysis is that quantitative data were normalized to the lesion size and reference values (see below). Moreover, the skeletonization was performed using our custom-made open-source software *Skelios*. Besides basic skeleton parameters, it yields detailed information about complexity and connectivity of the vascular network. The availability of the reference values (vascular network just before the partial amputation) gives the unique opportunity to reduce the bias and increase the reproducibility of the results. In addition, we introduce the optional 3-way correlative morphology approach to analyze and match the dynamic changes *in vivo* with their morphological substrate at ultrastructural level. According to our knowledge, there are no approaches reported that could provide such correlative microscopy data: *in vivo*-imaging down to single cells within the living organism, followed with the light and electron microscopy of the site of interest harvested at the chosen time-point.

In summary, we introduce an advanced, highly reproducible and non-embryonic angiogenesis assay using the zebrafish caudal fin. It is suggested for comprehensive quantitative and qualitative evaluation of the vascular alterations during normal vascular growth and under treatment conditions.

## Methods & Results

### Animal care and conditions

**Fig 1:**
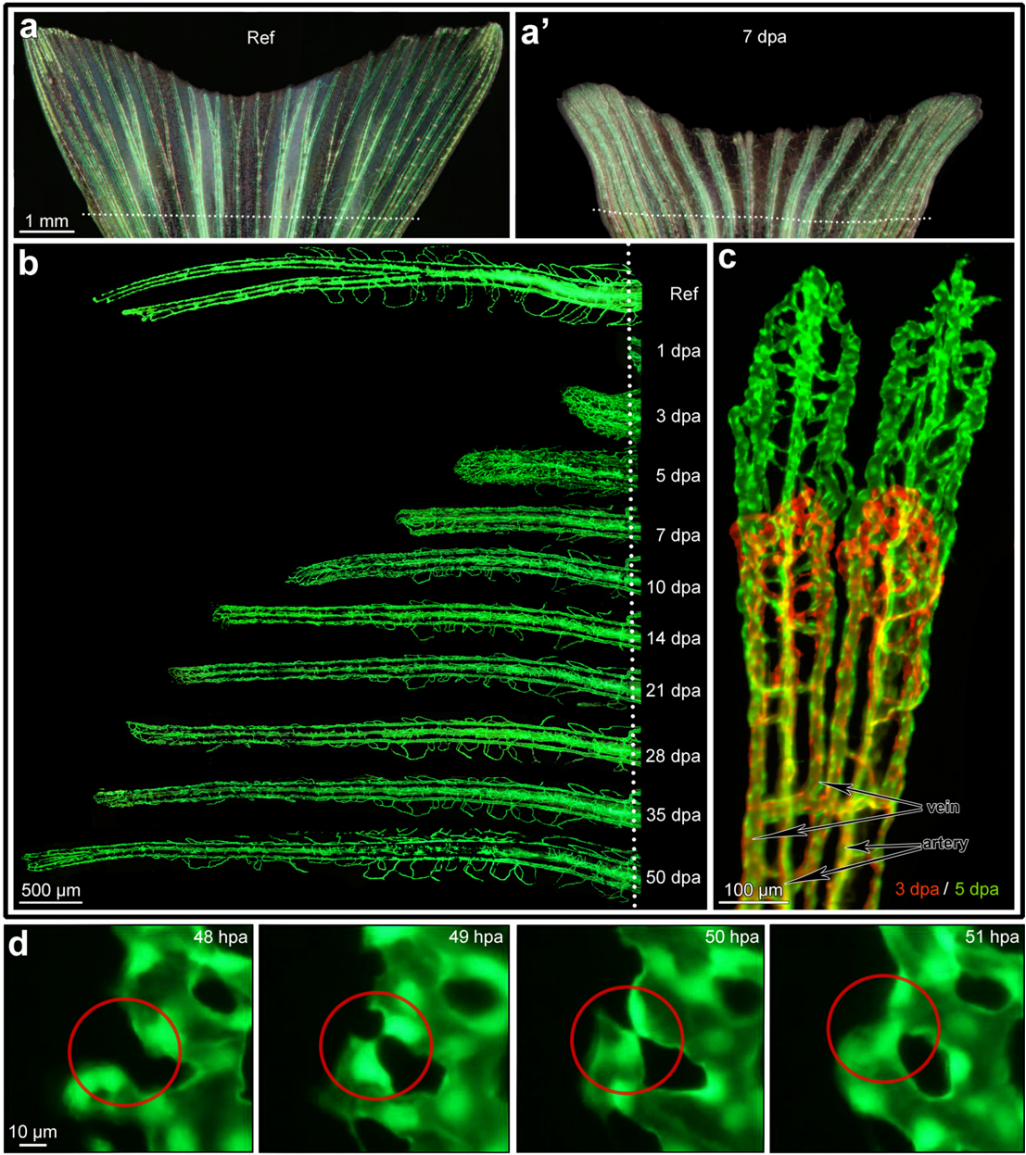
In vivo monitoring of the angiogenic process during fin regeneration. **Panel** “**a”** displays the reference image of caudal fin (just before amputation) taken with both normal bright and fluorescent reflected light. The vessels appear green (eGFP in Tie-2 positive endothelial cells). **Panel”a‘”** displays a representative image of the regenerated caudal fin at 7 dpa (days post amputation). The dotted line indicates the amputation level. **Panel** “**b”** displays the vascular regeneration process at higher resolution at example of one fin ray. Starting from 10 dpa the vasculature starts to resemble the reference vascular pattern before amputation (Ref). The red-green image in panel **“c”** demonstrates the outgrowth of the vasculature between 3 dpa (red) and 5dpa (green): one can easily follow the formation of every single vessel segment in dynamics. Arteries situated in the central part (core) of the ray (one per ray) can be clearly distinguished from the veins (2 per ray) situated aside from the ray core (see markings in **c**). **Panel “d”:** high resolution *in vivo* time lapse images demonstrating the fate of single capillary sprouts within 4 hours. Left image shows the vessels at 48 hours post amputation (hpa), already one hour later opposing sprouting tips are approaching each other. By 50 hpa, the opposing vessel walls seem to be already attached. At 51 hpa the new vascular segment is formed and already perfused. Endothelial cell nuclei are fluorescing intensive green.

This study was carried out in strict accordance with the Swiss Animal Welfare Act and Swiss Animal Welfare Ordinance. The study protocol was approved by the Bernese Cantonal Animal Welfare Commission and the corresponding permit was then issued by Bernese Cantonal Veterinary Office (permit number: 125/12). All manipulations were done under anaesthesia with tricaine, and all efforts were made to minimize suffering. The fish were monitored once daily on swimming behavior, body weight (estimated by eye), crooked vertebral column and overall appearance. No fish showed signs of pain or distress during the experiment. There were two deaths during the experiments in the long-term follow-up (50 dpa)–most probably due to the prolonged anesthesia time. There were another 2 deaths in the highest concentration of the CoCl2 group. The concentration possibly exerted toxic side effects. The *Tg(Tie2*:*EGFP)*^*s849*^ zebrafish line was obtained from the Zebrafish International Research Center. During pilot experiments, we could not detect any significant difference between *this line* and the *Tg(fli1a*:*EGFP)*^*y1*^. Thus, any of these lines can be used in the introduced assay. *Tg(Tie2*:*EGFP)*^*s849*^ zebrafish aged 10–14 months were used to visualize endothelial cells *in vivo* (Fig 1). Throughout the experimental period, zebrafish were kept in a well-lit incubator with a 14h/10h day-night-cycle. The temperature was set to 30°C and was continuously monitored. The slight increase in temperature compared to the standards of other laboratories (28.5°C) leads to faster regeneration and, therefore, earlier appearance of drug effects. A fully automated stereomicroscope (Leica Microsystems M205FA) with a mounted Canon EOS 5D Mark II camera and an XY-table was used for the dynamic follow-up. Overlapping images were merged and superimposed using Adobe Photoshop CS5 (Fig 1). Prior to amputations of the caudal fin as well as during the observation periods, zebrafish were anesthetized using 0.04% MS-222 (tricaine). The caudal fin amputation (approx. 50% lesion size) was performed using a razor blade and perpendicular to the cranio-caudal axis of a fish.

### Monitoring of the angiogenic process

**Fig 2:**
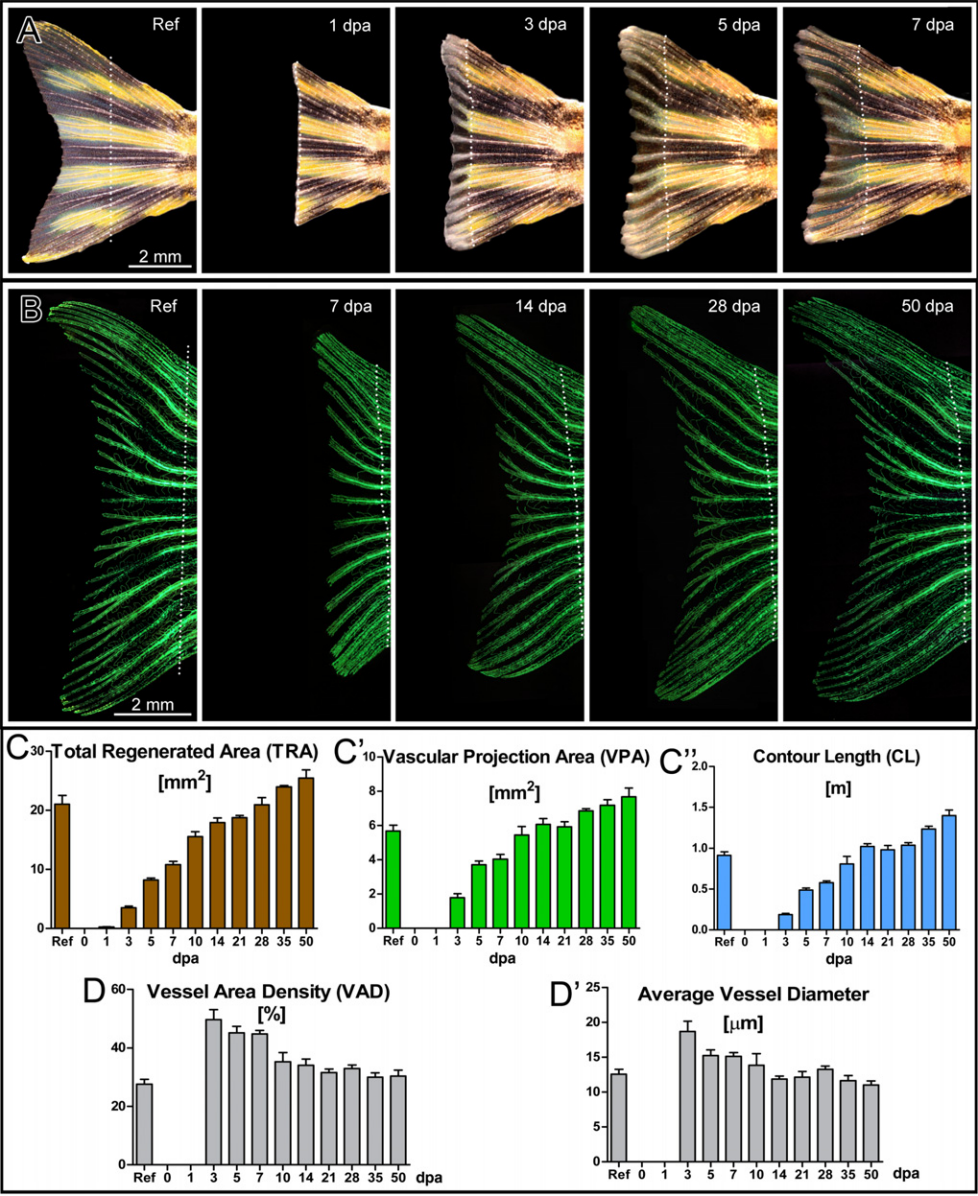
Regeneration of the zebrafish caudal fin and time courses of total regenerated area (TRA), vascular projection area (VPA), vessel wall (contour) length (CL) and their derivatives during regeneration. The process of caudal fin regeneration at multiple time points from reference to 50 dpa: the upper row (A) represents bright field images at reference, 1, 3, 5, and 7 dpa. The second row (B) contains fluorescent images at reference, 7, 14, 28, and 50 dpa. Such images allowed quantitative assessment of time courses of multiple general and vascular parameters as presented in the graphs C-C´´, D and D´. At least 50% of the cut fin (TRA) is regenerated within the first 7 days (C) and the rapid angiogenesis between day 3 and 7 leads to increase of the vessel area values almost up to the reference level (C´). The levels of contour length reached reference level at 10–14 dpa. Vessel area density (D) as well as average vessel diameter (D´) were remarkably higher in comparison with the reference up to 7 dpa.

In a first step, regeneration of the zebrafish caudal fin was documented over 50 days post amputation (Fig 1B). In this time period, the fin vasculature reached the vascular pattern and vascular parameters documented prior to fin amputation, which served as the reference for complete regeneration. The model offers the possibility for repetitive observations of the same animal at multiple time points, covering days or even weeks (Figs 1B, 1C and 2B). Highly dynamic processes, such as endothelial sprout formation, consecutive tip cell fusion and finally perfusion of the new vascular segments, can be closely monitored over few hours (Fig 1D).

The high reproducibility of vessel regeneration, in combination with repetitive observation of the same vessels over longer periods of time, offers reliable high quality *in vivo* follow-up and leads to a dramatic reduction in the number of animals needed for a study.

### Stereological assessment

Stereology describes methodological approaches for structural quantification in microscopy [24]. With respect to our quantification goal, the particular structure of the zebrafish caudal fin can be considered as a near-planar 2D-system with the supplying artery and vein pair per ray (Fig 1C) and connecting capillaries, too (see also [23]). Vascular exchange surface was determined as the area of the outer surface of the vasculature. Vascular exchange surface and vessel diameter were assessed assuming that blood vessels are round (cylindrical) structures.

Three variables describing the whole zebrafish caudal fin, namely, “total regenerated area” (TRA), “vascular projection area” (VPA), and “contour length” (CL) of the blood vessels were introduced (Fig 2C–2C´´ and S1 Table). TRA designates the area of regenerated caudal fin regardless of its vascularization. VPA is the area of all vessels within the regenerated part as projected on fluorescence images. The commonly used derivative of the two mentioned parameters is the vessel area density (VAD): VAD = VPA/TRA. VAD represents the density of the vessel projection area within the regenerated tissue (Fig 2D). Contour length (Fig 2C´´) is the estimated length of the vessel projection contour (see S1 Fig). In order to re-ensure the functionality of the fin, i.e. the fish‘s ability to swim, the fin tissue had to regenerate rapidly. Indeed, TRA increased more than 1.5 mm^2^ per day (1.55 ± 0.17 mm^2^) during the first week of regeneration (Fig 2C). Vessel growth was not observed before 1 dpa when first anastomoses between the distal tips of the artery and the veins began to form [23]. Already at 3 dpa, a tightly connected vascular plexus was present, represented by a rapid increase in VPA and CL. This plexus is formed through very active sprouting angiogenesis (Fig 1D). As a result, vessel area density (VAD) rose to 50% (0.50 ± 0.07) at 3 dpa. Thereafter, VAD gradually decreased to the reference value. Noteworthy, the normalization to the reference value is critical since the absolute values increase proportionally to the size of the lesion and should not be directly compared (see S2 Fig). TRA, VPA and CL all regenerated to roughly 100% of the reference at 14 dpa. These findings support the idea of previous studies that fin regeneration terminates at 14 dpa. Still, we expected a longer time period for restoration of the vasculature to occur (see below). Regenerative outgrowth continued also after reaching reference levels (i.e. 100%). This phenomenon is due to the ongoing growth of the fish and its fins also during the adulthood.

Taken together, it becomes evident that the most substantial part of angiogenesis and tissue regeneration takes place before 7 dpa (Fig 2). This fact is reflected by the dramatic increase in all parameters investigated: TRA, CL, VPA and VAD (Fig 2C–2C´´). Therefore, we decided to set the duration of our “detailed angiogenesis assay” (DAA) to 7 days (see also S3 Fig).

### Skeletonization yields information about connectivity of the vascular network

**Fig 3:**
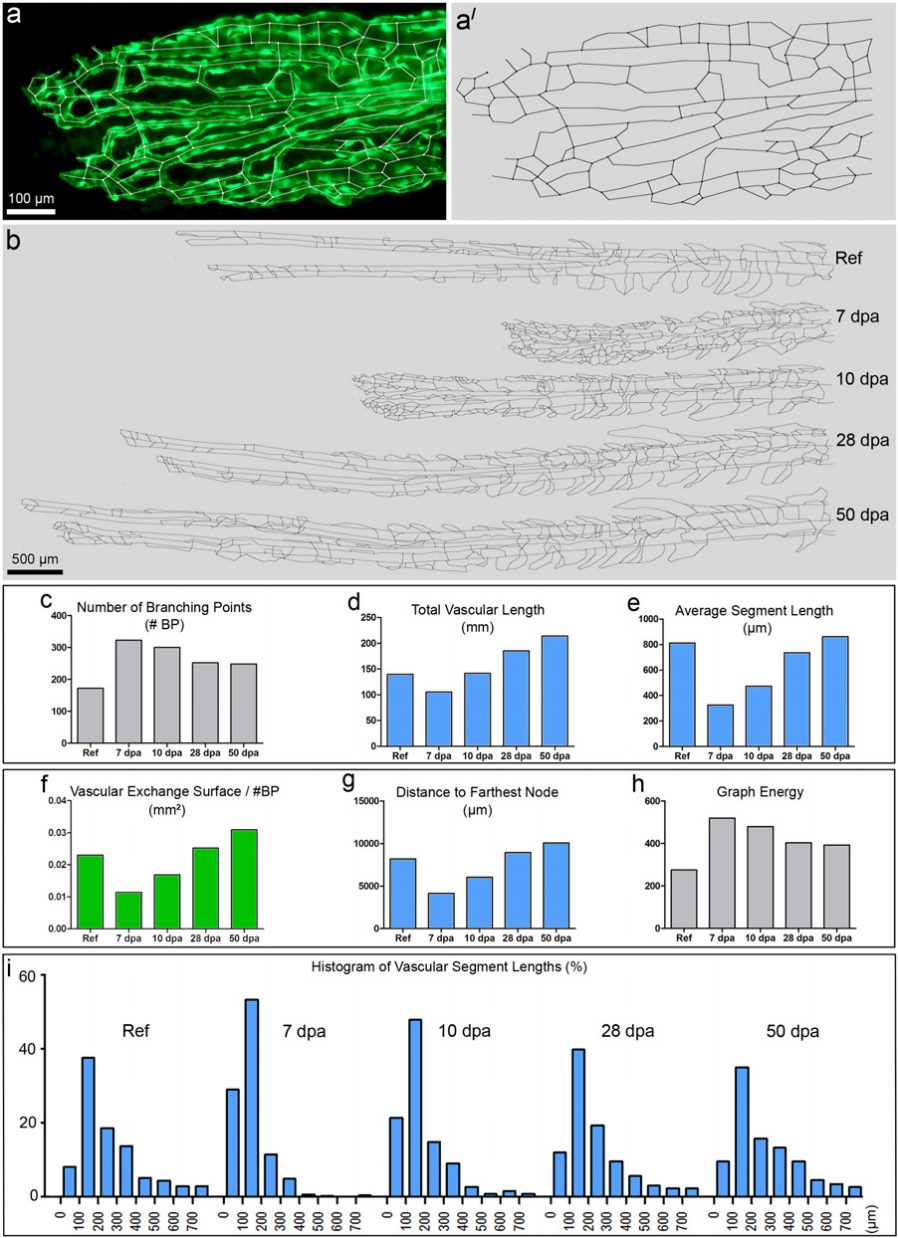
Skeletonization of the vasculature of the 4^th^ fin ray and its quantification. **a** displays the distal vasculature of the 4^th^ fin ray with overlaid skeleton. ***a*’** displays the same skeleton without the original image. ***b*** represents the skeletons of regenerated parts of the same 4^th^ ray at different time points (reference, 7, 10, 28 and 50 dpa). The graphs in ***c-i*** display dynamics of the parameters used for quantitative analysis of the skeletons at different time points: number of branching points (***c***), total vascular length (***d***), average segment length (***e***), and vascular exchange surface per branching point (***f***), distance to farthest node (***g***), graph energy (***h***). The histograms in ***i*** showed a clear left shift at 7 dpa and 10 dpa due to the higher number of branching points and respectively shorter vascular segment lengths.

In order to investigate the maturation status and complexity of the vascular network, we again took advantage of the 2D-structure of the caudal fin and introduced vascular skeletonization. For skeletonization, the area of interest has been reduced to the fourth dorsal fin ray (S4 Fig). It is one of the fastest regenerating fin rays and often lies between two stripes of melanophores, which may shield fluorescent signal [25]. Skeletonization itself was performed using our self-made skeletonization program *Skelios*. *Skelios* works on a user-driven approach and is available as an open-source program (http://doi.org/10.17605/OSF.IO/3GRW9). An example of the vasculature and the obtained corresponding skeleton is presented in Fig 3A and 3A’. Skelios software analyses the skeleton-derived graph (i.e. a model representation for depicting pairwise relations) and allows determining over twenty vascular parameters like the number of branching points, the total length of the vascular network (total vascular length = TVL), average and individual segment lengths, vascular exchange surface per branching point, distance to farthest node, graph energy etc. These parameters have been followed during the entire time of vasculature regeneration (Fig 3B–3I).

As previously mentioned, all stereological variables (VPA, CL and TRA) reach ≈100% of the reference value within 10–14 days. Although this may be true, the vasculature does not yet appear properly restored. Strikingly, the number of branching points (#BPs) in the 4^th^ ray at 10 dpa was still 174.42% of the reference value (Fig 3C). In contrast, the total vascular length was fully restored (Fig 3D). As a result, the average length of a vascular segment was shorter by 42% (Fig 3E). The latter fact was respectively reflected in the histograms, which reveal the distribution of all vascular segments during the regenerative process (Fig 3G). In early stages (<10 dpa), the vascular segment length distribution was shifted to the left, thus representing a high number of short segments. In later regenerative stages (>10 dpa), the distribution converges to the reference situation.

Although VPA and CL reached reference level at 10–14 dpa (Fig 2C´ and 2C´´), the vascular network appeared non-optimized, underdeveloped, and lacked the characteristic vascular pattern in comparison with the reference network, i.e. regeneration, and namely, restoration of the vasculature, was not completed. The vascular network was subsequently optimized. Only at 28 dpa, VAD, average vessel diameter (Fig 2D and 2D´), vascular exchange surface per branching point (Fig 3F), and average segment length (Fig 3E) returned to the reference level. Vascular *exchange surface per number of BPs* reflects the functional derivative of VPA, i.e. the area accessible for oxygen and nutrients to diffuse, normalized to the number of branching points. As a result, it is a very suitable variable for monitoring vascular maturation. The *distance to farthest node* (Fig 3G) represents the shortest path a blood cell has to make in order to reach the farthest (most distal) point of the regenerating vasculature.

The newly introduced parameter *graph energy* (Fig 3H) characterizes the connectivity of the vascular network. The graph energy rose from theoretical chemistry where its value is associated with the stability of molecules and more generally between connected components. Briefly, the larger the graph energy, the more connected is the graph (for more details see S1 Text).

The process of caudal fin tissue regeneration takes roughly two weeks. At least another two weeks are needed to complete restoration of the vasculature. The vasculature was considered as restored/mature when all the vascular parameters (including the advanced ones) reached at least 100±20% of the reference level. To conclude, the combination of stereology and skeletonization was necessary to determine whether regeneration is complete.

### Time-efficient Detailed Angiogenesis Assay (DAA) and evaluation of an anti-angiogenic compound (PTK787)

Combining stereology and skeletonization is a powerful strategy to investigate angiogenesis in a dynamic way, from its initiation to pruning and maturation of the vascular tree. The pro- or anti-angiogenic potential of applied compounds is often the central aspect of preclinical and basic research studies. As demonstrated above, angiogenesis is most pronounced within the first 7 days post amputation. Based on this fact, we introduced the “detailed angiogenesis assay”, which lasts up to 7 dpa (S3 Fig). The DAA covers the period of most dramatic angiogenic events (Fig 2C–2C´´) for a time-efficient but thorough evaluation of drug effects. The two most important angiogenic modes, sprouting and intussusceptive angiogenesis are both present within this period (Fig 1). DAAs need no more than 7 days of animal care: 24 hours after fin amputation, the target drug is applied into the tank water. Thereafter the tank water is changed every other day and the compound added (S4 Fig). Throughout the experimental period, a maximum of two zebrafish were maintained per one 0.7 L plastic tank with a minimum of 200 ml water (+ drug solution). They were fed twice per day. The risk of endothelial cell damage due to extensive fluorescent exposure is minimized because the fish are given 48h periods of rest. Besides the reference, the suggested observation time-points are: 3, 5 and 7 dpa. We tested our method with a well-known VEGFR inhibitor, PTK787, at 4 different concentrations, namely, 50, 100, 250 and 500 nM (final concentration in the tank water).

The VEGFR inhibitor used here (PTK787, Novartis), is a small molecule protein kinase inhibitor that inhibits angiogenesis. In *in vitro* assays with HUVEC cells it is most potent against VEGFR-2 (KDR) IC_50_ = 37 nM as well as against VEGFR-1 (Flt-1) IC_50_ = 77 nM [26]. At higher concentrations, other tyrosine kinase members like PDGFR-β, c-KIT, and c-FMS are also inhibited. However, it does not inhibit EGFR, TIE-2 or intracellular kinases [26]. PTK787 drastically reduces vessel density in tumor tissues through inhibition of angiogenesis [26;27].

**Fig 4:**
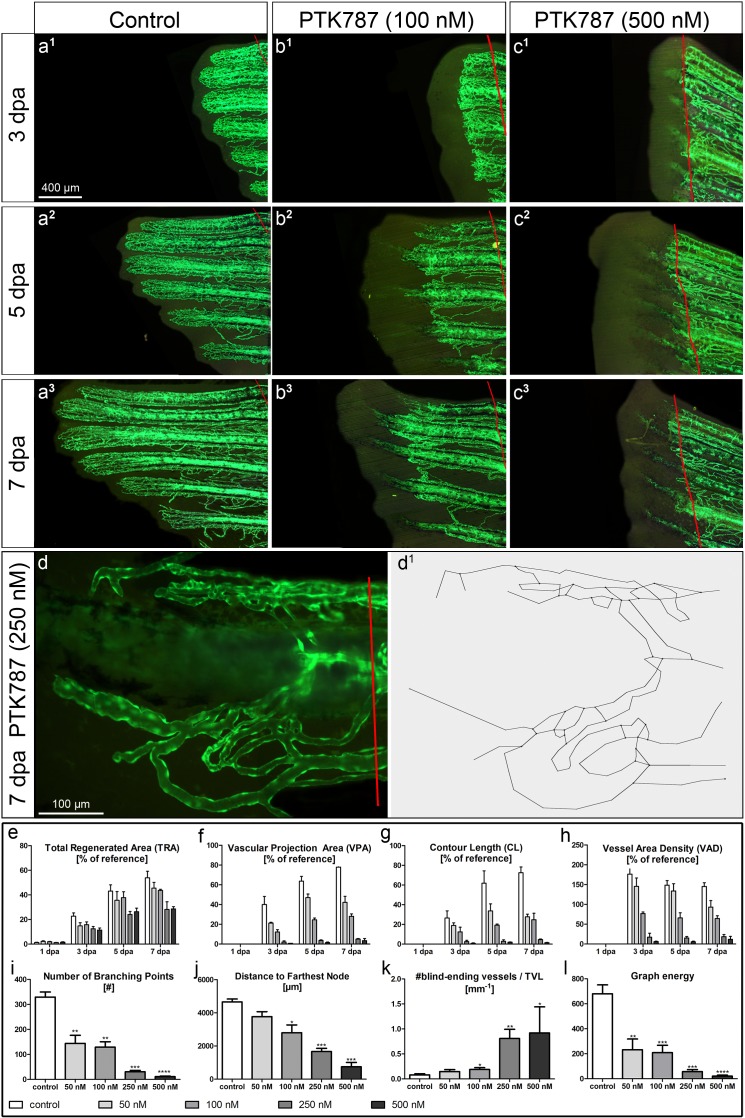
**DAA: Assessment of the anti-angiogenic effects of PTK787 at different concentrations 50, 100, 250, 500 nM: a-c** Fluorescent images of regenerating caudal fins at 3 (**a**^**1**^**-c**^**1**^), 5 (**a**^**2**^**-c**^**2**^) and 7 dpa (**a**^**3**^**-c**^**3**^) under 3 different conditions: vehicle control (left column), 100 nM (middle) and 500 nM PTK787 (right column). PTK787 clearly inhibits regenerative vascular outgrowth in a dose-dependent manner as confirmed by the quantitative assessment (see graphs ***e-l***). Based on the high-magnification images of the single fin rays at 7 dpa (example in **d**), the skeletons of the regenerating vasculature were obtained (**d**^**1**^). Advanced analysis of the vasculature of the 4^th^ ray at 7 dpa allows quantitative differentiation between multiple concentrations of PTK787. Although tissue regeneration was remarkably inhibited at 5 and 7 dpa at 250 and 500 nM (***c***^***2***^**, *c***^***3***^ and ***e***): at 50 and 100 nM of PTK787—no such prominent effects were observed (***b***^***2***^**, *b***^***3***^ and ***e***). VPA (***f***), CL (***g***) and VAD (***h***) showed dose-dependent inhibition with the difference between two lowest concentrations decreasing towards day 7. Four advanced (skeleton-based) parameters are presented in the lower row of graphs (***i-l***). Number of BPs (***i***) clearly distinguishes between lower and higher concentrations of PTK787, with the distance to the farthest node (***j***) being most informative/reliable for differentiation between all four concentrations applied. The number of blind-ending vascular segments per total vascular length (***k***) turned out to be capable of distinguishing the 4 concentrations applied. Interestingly, the newly introduced parameter graph energy (***l***) describing the connectivity of the vascular network detected highly significant differences between all the applied concentrations and the reference. *—p<0.05; **—p<0.01; ***—p<0.001; ****—p<0.0001.

PTK787 clearly inhibited angiogenesis and regeneration in a dose-dependent manner (Fig 4). The described stereological approach revealed that tissue regeneration (TRA) was remarkably inhibited only at 250 and 500 nM. No major inhibitory effects were observed at 50 and 100 nM. The parameters VPA, CL and VAD showed dose-dependent decline with the difference between the two lowest concentrations decreasing towards 7 dpa. By the use of skeletonization of the 4^th^ fin ray at 7 dpa with the following construction of the **skeleton-derived graph** (branching points “become” graph nodes) and its analysis, we could clearly discriminate between the effects of the different PTK787 concentrations (Fig 4I–4L). In our application case, the number of BPs (Fig 4I) unambiguously distinguishes between lower and higher concentrations of PTK787. The distance to the farthest node in the obtained skeleton-derived graph (Fig 4J) and number of blind-ending vascular segments per total vascular length (Fig 4K) were most reliable for the differentiation between all four applied concentrations. The distance to the farthest node (most distal branching point) is mainly influenced by the outgrowth of potentially functional regenerated vasculature (the blind-ending vascular segments are not considered). Therefore, in case of PTK787, it is a good parameter for estimation of the vascular response; since it can clearly differentiate the outgrowth level of the vasculature by omitting the non-functional blind-ending vascular segments (see Fig 4B^2^, 4B^3^ & 4D and 4D^1^). Number of blind-ending vascular segments reflects the amount of vessels non-integrated into the vasculature, giving the idea about the disconnectivity in the vascular pattern. This number was normalized to the total vascular length for comparability between the vasculatures of different sizes. In case with PTK787 the number of blind-ending vessels per TVL was dose-dependent (Fig 4K), representing the increased disconnectivity within the regenerating vascular network.

The number of blind-ending vascular segments only indirectly influences the newly introduced parameter *graph energy*, which characterizes the connectivity of the vascular network. In case of PTK787 application, the graph energy (Fig 4I) displayed remarkable dose-dependent decrease, and proved to be another reliable parameter for differentiating between multiple concentrations of the compound.

**Fig 5:**
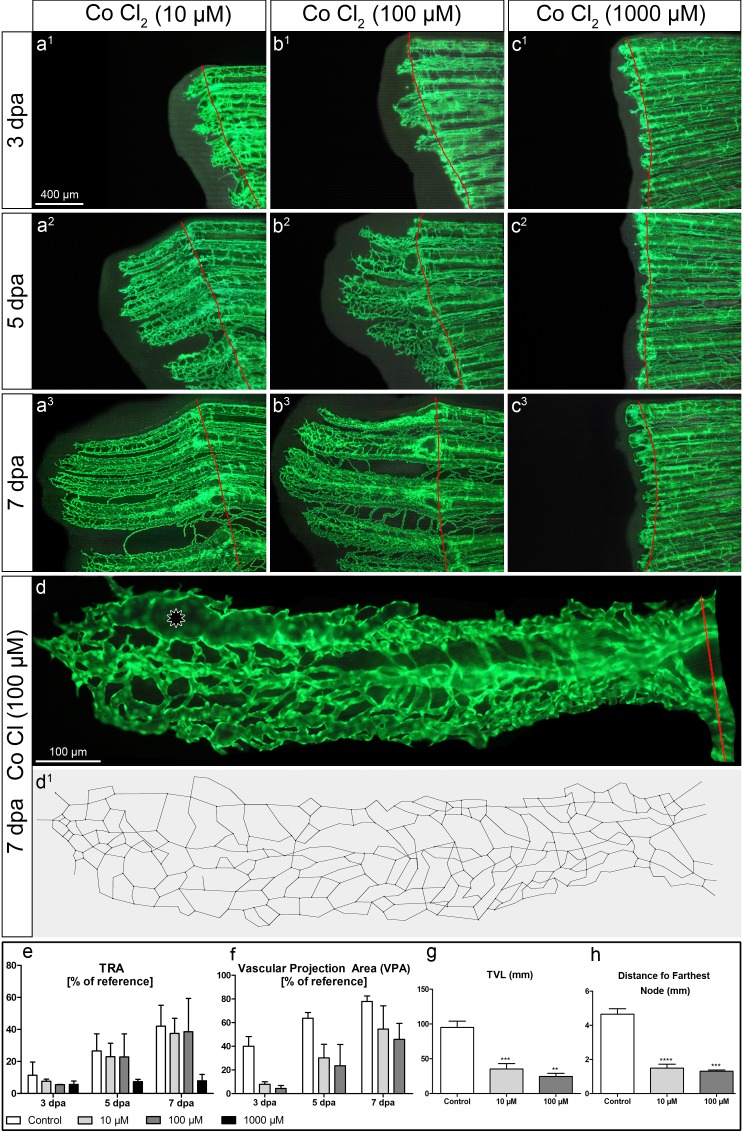
Assessment of effects of systemic cobalt chloride application at different concentrations 10, 100 and 1000μM. **a-c** Fluorescent images of regenerating caudal fins at 3 (**a1-c1**), 5 (**a2-c2**) and 7 dpa (**a3-c3**) under 3 different conditions: 10 μM (left column), 100 μM (middle) and 1000 μM of cobalt chloride (right column). Based on the high-magnification images of the single fin rays at 7 dpa (**d**), the skeletons of the regenerating vasculature at 7dpa were obtained (**d’**). The commonly used assessment values revealed no statistically significant differences (**e-f**). To focus on the effects of CoCl2-treatment in all applied concentrations, the controls are not displayed in this figure. The control fish used for the quantifications shown in the graphs (e-h) are the same as in Fig 4. TRA in 10- and 100 μM groups had no remarkable difference in comparison with the control values (**e**). At 7dpa, the vascular projection area could reveal some tendency to dose-dependent inhibition, but the difference was not statistically significant. That can be, at least, partially explained by the sporadic presence of dilated vessels (marked with an asterisk in **d**). The skeleton-derived parameters like total vascular length (TVL) (**g**) or distance to farthest node (**h**) are not influenced by dilated vessels and revealed statistically significant differences. *—p<0.05; **—p<0.01; ***—p<0.001; ****—p<0.0001. Remark: The mortality in the 1000μM-group was very high (only 2 out of 8 fishes survived till day 7)–that was the reason for not considering it for the advanced analysis.

In the case of another tested compound (Fig 5), CoCl_2_ (HIF-modulator), the stereologically assessed parameters like TRA and VPA failed to reveal a significant difference at 7 dpa between the 10 μM, 100μM and control groups (Fig 5E and 5F). The “failure” of VPA is exemplary and could be explained by sporadic presence of big dilated vessels (marked with *asterisk* in Fig 5D). Presence of such dilated vessels was not an obstacle for the advanced parameters like TVL (Fig 5G) or distance to farthest node (Fig 5H) to reveal highly significant differences between the treated and control groups.

### 3-Way Correlative Microscopy

**Fig 6:**
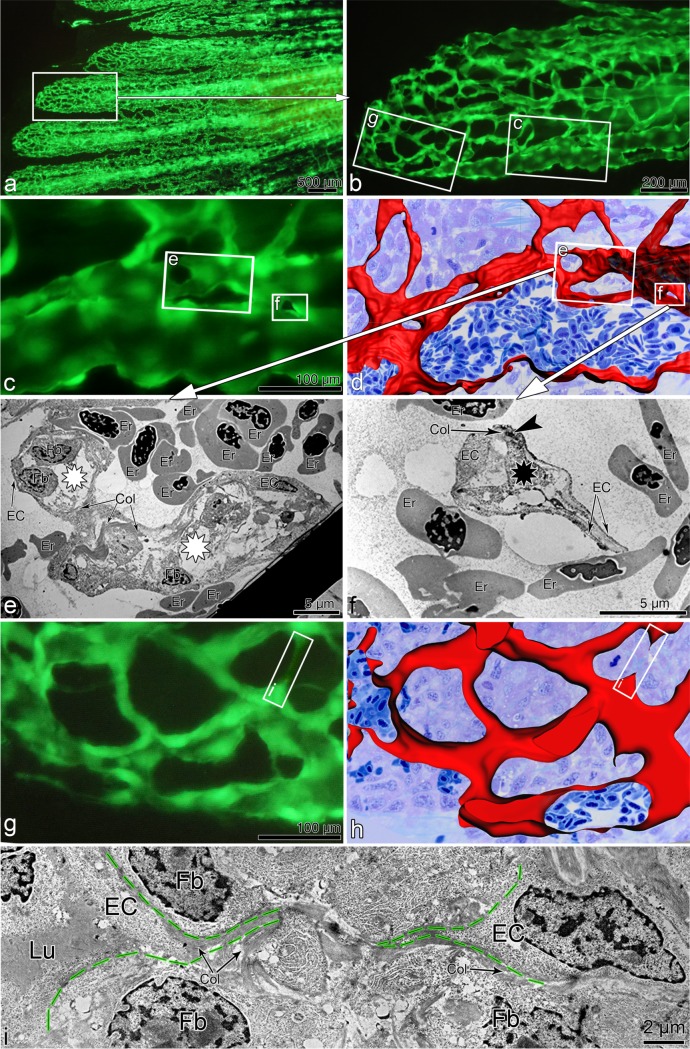
Correlative microscopy:Correlation of vascular alterations in vivo to their morphological substrate. in vivo. The *in vivo* fluorescent image in ***a*** shows the regenerating fin vasculature (5 dpa) at low magnification. ***b***, ***c*** and ***g*** zoom in the site of interest indicated by rectangles. Based on serial semithin sections, panels ***d*** and ***h*** display the 3D-reconstruction of the areas depicted in ***c*** and ***g*** respectively. **c-f**: proximal region of interest, mainly characterized by transluminal tissue pillars and meshes (sign of intussusceptive angiogenesis). Transmission electron micrographs in ***e*** & ***f*** demonstrate the pillars at ultrastructural level. Black asterisk (also smallest frame in **c** and **d**) indicates transluminal tissue pillars, while white asterisks show inter-capillary meshes (large pillars). One can also detect the cell-cell contacts in between the endothelial cells (dark area indicated with an arrowhead in ***f***). **g-i**: distal region with predominant sprouting mode of angiogenesis. Frames in **g** & **h** mark the sprout formation presented at ultrastructural level in **i**. Dashed green lines in **i** indicate the borders of fusing sprouts coming from opposing endothelial cells (*EC*): the sprouts are progressing along the collagen scaffolds. Er = erythrocyte, Col = collagen fibers, Fb = fibroblast, EC = endothelial cell, Lu–vessel lumen.

Angiogenesis in its full complexity can be investigated only by an approach combining several techniques. Here, we demonstrate the potential of the proposed model by matching the dynamic changes in blood vessels (or even single endothelial cells) extracted from *in vivo* observations with morphological data from light microscopic (LM) and transmission electron microscopy (TEM) (Fig 6). We repeatedly observed the regenerating vasculature, focusing at multiple sprouts in the distal vascularization front (hardly perfused) and transluminar pillars in the proximal (perfused) regions. Thereafter, the sites of interest (Fig 6A–6D) were harvested at 5 dpa, fixed in 2.5% glutaraldehyde and processed for serial alternating semithin/ultrathin sectioning (2 semithin sections at 400 nm followed by 3 ultrathin at 70 nm thickness) (see also S5 Fig for more details). The series of semithin sections were then stained with toluidine blue and the images taken at low resolution (x20) first. The images were processed into 3D-stacks and analyzed using Imaris Software (Bitplane AG, Zurich, Switzerland) (Fig 6E & 6G). The vessels were specially marked with red color for their easier tracking. Then, the projections of obtained 3D reconstructions were superimposed with *in vivo* images in order to understand the orientation and coarsely localize the site of interest on the semithin sections. Once the site of interest is localized, in the second step, the images of the potential site of interest were taken from semithin sections at higher magnification (usually x63) and again the 3D-reconstruction with vessels marked was done. This second step is critical for fine matching and precise 3D-localization of the cells and structures of interest on the ultrathin sections, which were then observed in a Philips EM-400 transmission electron microscope (TEM). The obtained TEM micrographs (Fig 6E, 6F & 6I) gave deeper insights into the morphological substrate and tissue components involved: e.g., transluminar pillars containing a collagen core surrounded by endothelial cuff with endothelial cell-cell contacts (Fig 6E & 6F). In another instance, we were able to show that sprouts use extracellular matrix scaffolds, which guide the outgrowth of tip cells (Fig 6I). Such morphological findings are crucial for the understanding of the processes involved.

### Statistical Assessment

For assessment of statistical significance we applied a 2-tailed *t*-test using the Prism GraphPad v5.04. The difference was considered statistically significant if p<0.05. Markings used in the graphs: *—p<0.05; **—p<0.01; ***—p<0.001; ****—p<0.0001; *n* means the number of biological replicates (not technical ones).

## Discussion

Angiomodulating drugs are developed to stimulate or inhibit angiogenesis during various pathological processes. The list of pathologies that could be treated with effective angiomodulating therapies is long and there are new pathologies joining in like the temporal lobe epilepsy, thus increasing the potential socio-economic impact of effective angiomodulating therapies [28].

There are plenty of *in vivo*-angiogenesis assays; in spite of this, the major limitation remains a comprehensible and swift quantification. Most researchers agree that reliable, reproducible and precise methodology to quantitatively assess angiogenesis is an imperative of high priority in the field of angiogenic research [5;6;29]. Embryonic models have the disadvantage of being embryonic. The known non-embryonic angiogenesis models have limited possibilities for *in vivo* investigations and, at the same time, for proper quantification. In addition, the rodent assays are expensive, technically demanding and it is hardly possible to follow the dynamical changes *in vivo* at a reasonable resolution. Moreover, they are more time-consuming and sometimes ethically questionable [5]. Often the investigating teams end up disappointed due to unsuccessful results, partially caused by study design, which could well be improved. This situation could be bettered by the use of another simple pre-clinical model prior going to the rodent or other mammal model systems. The zebrafish caudal fin regeneration model is a good candidate for this niche, and the utility of zebrafish as a model organism for chemical biology and adult angiogenesis has been rigorously demonstrated [19].

In spite of the applied model the proper assessment of angiogenesis is still a great challenge. For example, due to the fact that the fin rays curve towards the midline, in many instances the evaluation was done in rays 2–5 from the edges of the caudal fin, where the most consistent growth and vascularization is observed [19]. It turned out that every fin ray has its own regeneration speed–this can be explained by different size of the lesion caused to every single fin ray (see S4 Fig). Different sizes lead to different regeneration speeds (see S2 Fig). That is why it is important to focus on one fin ray and normalize the results, especially, if the quantitative assessment is desired.

One of the latest examples of quantitative assessment of angiogenesis compares vasculature regeneration based on the count of green pixels in the confocal image–the endothelial cells of transgene (fli1:eGFP) had a green fluorescence signal, but not always the lumen of the vessels (see Fig 6 in [30]). The data obtained, even though they might give an idea, do not reveal the number of vessels, their volume, surface, length, or any other relevant vessel parameters. Such an approach has difficulties with many possible aberrations: for example, it does not differentiate between many thin and a few dilated vessels (see Fig 5). Most importantly, quantitative data have not been correlated to the reference situation. An increased lesion size will result in a higher regeneration speed (S2 Fig) and hence introduce great bias. The implementation of the reference values (vascular network just before the partial amputation) gives the unique opportunity to significantly reduce the bias and increase the reproducibility and, therefore, credibility of the obtained results.

More meticulous was the quantification through measurement of vascularized and non-vascularized areas of the regenerated fin [19]. The latter information gives an idea on whether the newly formed blood vessels can follow the regenerative growth of the fin. In addition, the quantification methods applied this far are insufficient and lack a relation to the functionality. The lack of a proper quantification method for assessing angiogenesis makes the task of comparing multiple substances at various concentrations and their impact on vascular morphology unmanageable. Here, we suggest a quantification method, including the self-developed software tool *Skelios*, which allows using the caudal fin regeneration assay more efficiently than it has been performed previously. Besides stereological parameters like TRA, VPA, CL, VAD and average vessel diameter we introduce further parameters like vascular exchange surface per branching point, blind-ending vascular segments normalized to TVL, individual and average segment lengths, distance to farthest node, graph energy *etc* (*Skelios* provides over 20 parameters). These new parameters provide more profound information on the restoration level of the regenerating vasculature, i.e. whether the regeneration is actually complete. We would like to emphasize here on 3 following parameters allowing in probably most cases to distinguish between effects of different drugs or its concentrations. Firstly, distance to farthest node reflects the shortest path a blood cell has to make to reach the most distal point of the vasculature (most distal branching point) and is mainly influenced by the outgrowth of potentially functional regenerated vasculature. Omitting of the non-functional blind-ending vascular segments enables precise estimation of the outgrowth level of the functional vasculature. Secondly, number of blind-ending vascular segments per TVL gives the idea about the frequency of the vascular segments that are not integrated into the vasculature and cannot be perfused. The higher is this number the more disconnected (in this sense also immature) is the vasculature. Thirdly, *graph energy* represents the connectivity of the graph: the more compact and connected the network is, the higher is the graph energy. Therefore, at early stages of vascular regeneration (plexus formation) it is augmented and is decreasing afterwards while the vasculature undergoes remodelling and optimization (incl. by means of pruning). The three mentioned parameters turned out most effective for distinguishing between different concentrations of angiomodulating drugs.

Here, we have shown that restoration of the zebrafish caudal fin vascularization takes more time than the vast regeneration of blood vessels. Still, the substantial part of angiogenesis takes part within the first 7 days post amputation. Based on this finding, we introduce the time-efficient “detailed angiogenic assay” providing profound analysis within a rather short timeframe. The entire DAA takes no more than 7 days to perform, including the quantitative analysis using stereological approach as well as *Skelios* software. The working time needed is considerably less than 2 days (8–12 hours per 5 fish including skeletonization and eventual image post-processing: two full-time employees should be able to analyze around 40–50 fish per week). In other words, with n = 4–5 per compound (one group with single concentration) two full time employees are able to analyse 10 compounds per week or, e.g., only 2 compounds but at five different concentrations. With experience, this number may increase up to 100 fish per working week per two employees. By numbers over 100 fish per week one may consider installing another stereomicroscope. The time and manpower needed for optional correlative microscopy with the involvement of 3D reconstruction based on serial semithin sections and transmission electron microscopy is not included in the mentioned time line. All tasks related to the 3-way correlative microscopy are highly dependent on specialized staff and equipment.

Another great advantage of the DAA is its flexibility. Treatment periods, water temperature or other parameters can be adapted to the needs of the fish or the drug solutions (e.g., half-life). The custom-made skeleton analysis software provides more sophisticated parameters of the vascular network and is, therefore, capable of clearly distinguishing between multiple concentrations of the drug applied or even of different drugs at various concentrations.

### Limitations of the assay

At its present stage of development, the assay cannot be used for automated compound screening due to practical issues. Some steps are still user-driven. Furthermore, the need for anesthetization before visual analysis makes automation difficult [19]. Therefore, we can hardly imagine it as a high throughput screening procedure. All tasks related to the optional 3-way correlative microscopy are highly dependent on specialized staff and equipment. It is time-consuming and it cannot be assumed that a laboratory or company will use this as a routine-investigation. Hence, it should only be selectively applied to characterize drug effects in the very detail.

### Scope of application

It may be a very useful second-tier system to evaluate efficacy of drug candidates that have already shown angiomodulating activity in embryonic zebrafish or other biological assays, before investing time and effort on extensive analysis in multiple biological systems [19;20].

### Conclusions

The angiogenic process can be investigated in its full complexity only *in vivo*. Besides challenging quantification, correlative microscopy down to the ultrastructural level has not yet been introduced in such experimental models. The existing correlative microscopy-approaches are limited to observation of fixed material under light/fluorescence microscope with its further investigation in the electron microscope. According to our present knowledge, there are no approaches reported that could provide such correlative microscopy (*in vivo*-LM-TEM): *in vivo* imaging down to single cells within the living organism, followed with the light and electron microscopy of the site of interest harvested at the chosen time-point. Here, we introduce the optional 3-way correlative morphology approach to dynamically follow the morphological alterations in the vasculature *in vivo* for short time (minutes or hours) and/or over many weeks with their subsequent visualization at the ultrastructural level. Hence, it is a way to overcome one of the most critical disadvantages of the *in vivo* angiogenesis assays. Introduced DAA with optional 3-way correlative morphology and advanced quantification bring the caudal fin regeneration assay at a qualitatively new level, making it a very promising *in vivo* angiogenesis assay.

## Supporting Information

S1 Fig Stereology. (DOCX)

S2 Fig Reference concept: Influence of lesion size. (PDF)

S3 Fig Experimental setup of detailed angiogenic assay (DAA). (PDF)

S4 Fig Identification and quantification of individual fin rays. (PDF)

S5 Fig Scheme and detailed description of the 3-way correlative microscopy. (PDF)

S1 Table Formulary. (PDF)

S1 Text Info on the graph energy. (PDF)
