# Supplementary material for: Zebrafish Caudal Fin Angiogenesis Assay—Advanced Quantitative Assessment Including 3-Way Correlative Microscopy
Source: PLoS One. 2016 Mar 7;11(3):e0149281. doi: 10.1371/journal.pone.0149281 (PMC4780710; doi:10.1371/journal.pone.0149281)
Supplement: S1 Fig — (DOCX) [file pone.0149281.s001.docx]

**S1 Figure.** *Stereology*

**(TRA) Total Regenerated Area:**

The amputated area is indicated in red. Test grids with known square size (point associated area) were randomly overlayed on the fin. Points falling on the area of interest were summed (ΣP).

$$TRA= N_{p}*\left( \frac{area}{point} \right)$$

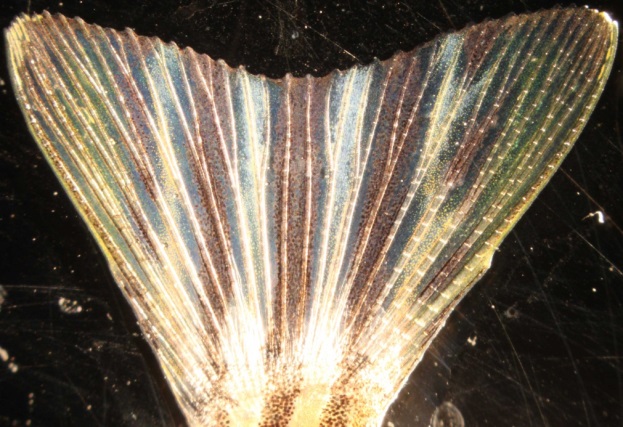

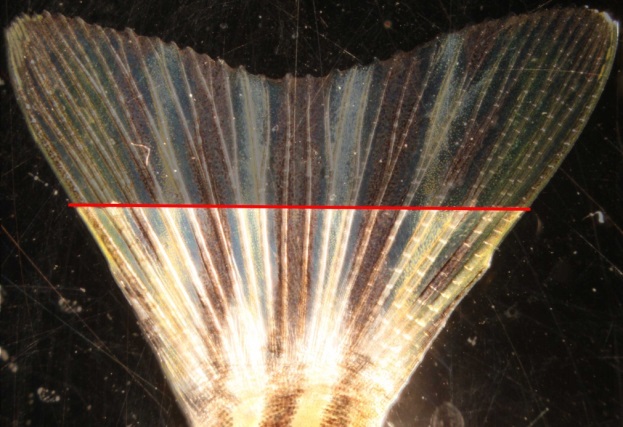


**TRA: Total Regenerated Area**


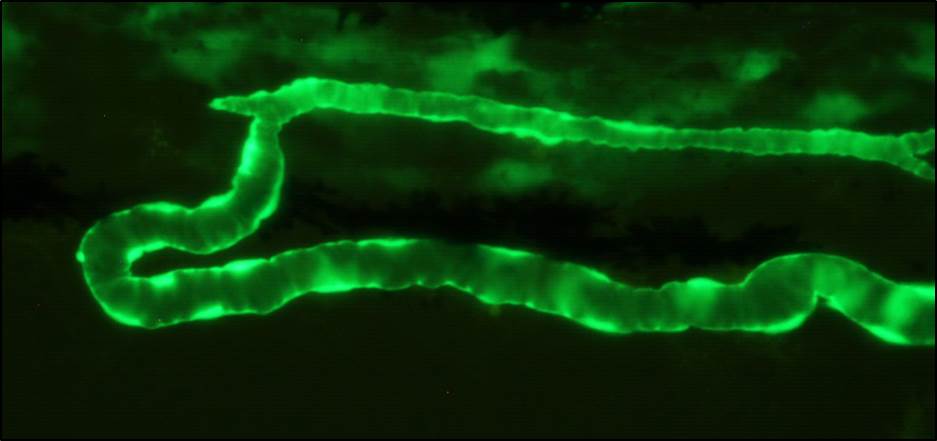


**VPA: Vascular Projection Area**


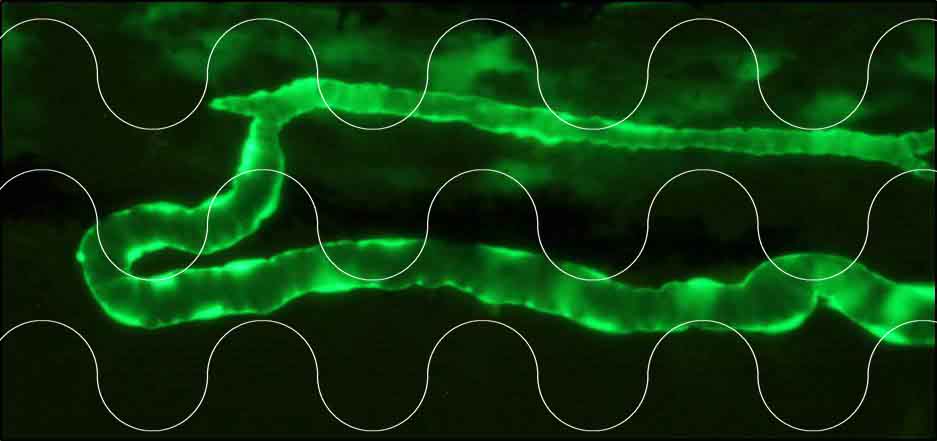


**CL: Contour Length**

**(VPA) Vascular Projection Area:**

VPA can be quantified for this simple vascular loop using the same formula as for TRA.

$$VPA= N_{p}*\left( \frac{area}{point} \right)$$

**(CL) Contour Length:**

In order to determine CL, circular test lines were virtually overlayed. ΣP is the number of times the test line crosses the contour of the blood vessel. dL represents the distance between the test lines.

$$CL= N_{p}*\frac{\pi}{2}*dL$$
