# Supplementary material for: Zebrafish Caudal Fin Angiogenesis Assay—Advanced Quantitative Assessment Including 3-Way Correlative Microscopy
Source: PLoS One. 2016 Mar 7;11(3):e0149281. doi: 10.1371/journal.pone.0149281 (PMC4780710; doi:10.1371/journal.pone.0149281)
Supplement: S2 Fig — (PDF) [file pone.0149281.s002.pdf]

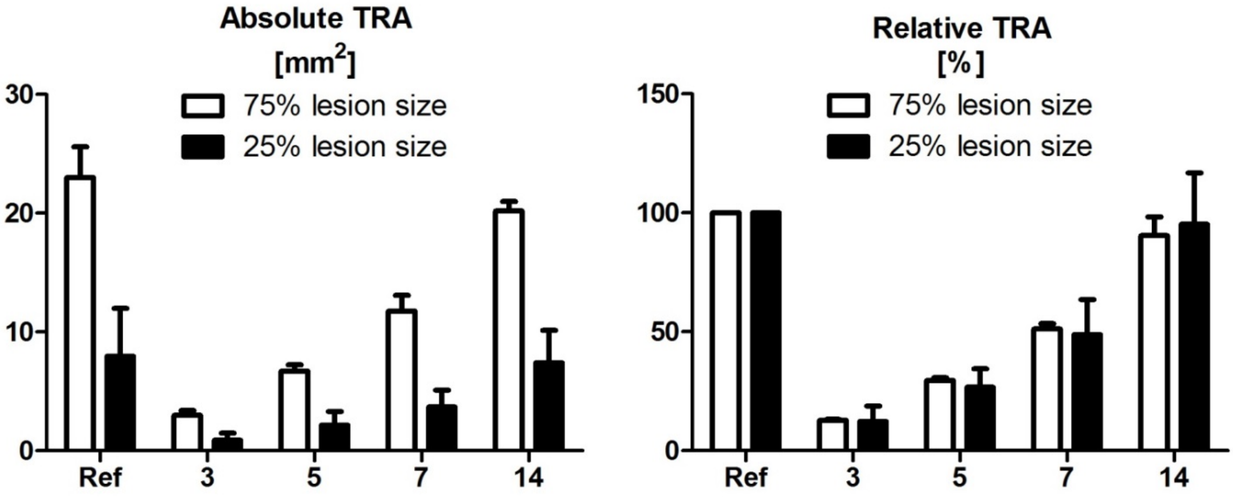

S2 Figure. *Reference concept: Influence of lesion size (n = 4)*

The caudal fin was partially amputated at either approximately 25% or 75%. This experiment revealed the importance of the reference concept. The smaller the lesion size, the smaller the regenerated area and its growth speed and vice versa (left panel). However, all results are directly comparable if expressed in relative values (right panel). Noteworthy, standard deviations were consistently smaller when bigger lesion sizes were applied.
