# Supplementary material for: Zebrafish Caudal Fin Angiogenesis Assay—Advanced Quantitative Assessment Including 3-Way Correlative Microscopy
Source: PLoS One. 2016 Mar 7;11(3):e0149281. doi: 10.1371/journal.pone.0149281 (PMC4780710; doi:10.1371/journal.pone.0149281)
Supplement: S3 Fig — (PDF) [file pone.0149281.s003.pdf]

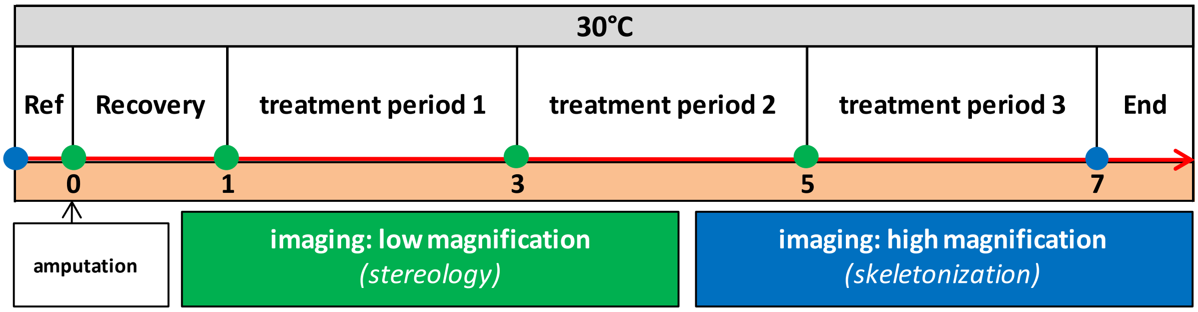

S3 Figure. *Experimental setup of detailed angiogenic assay (DAA).*

Zebrafish were first imaged right before amputation of the caudal fin (Ref). For pro- and anti-angiogenic drug solutions, one day of recovery for the fish is advisable to let anastomoses between arteries and veins at the regeneration front to be properly formed. For PTK787, water and drug solution were exchanged at 1, 3, and 5 dpa, thus leading to three treatment periods of equivalent concentrations. In order to reduce damage following excessive fluorescent exposure, the dynamic follow-up was performed using imaging at low magnification (50x). These images were analyzed using stereological approach. High magnification images (200x and more) were acquired only at the reference time point (before amputation) and at 7 dpa. These images were used for skeletonization and advanced analysis.
