# Supplementary material for: Zebrafish Caudal Fin Angiogenesis Assay—Advanced Quantitative Assessment Including 3-Way Correlative Microscopy
Source: PLoS One. 2016 Mar 7;11(3):e0149281. doi: 10.1371/journal.pone.0149281 (PMC4780710; doi:10.1371/journal.pone.0149281)
Supplement: S4 Fig — (PDF) [file pone.0149281.s004.pdf]

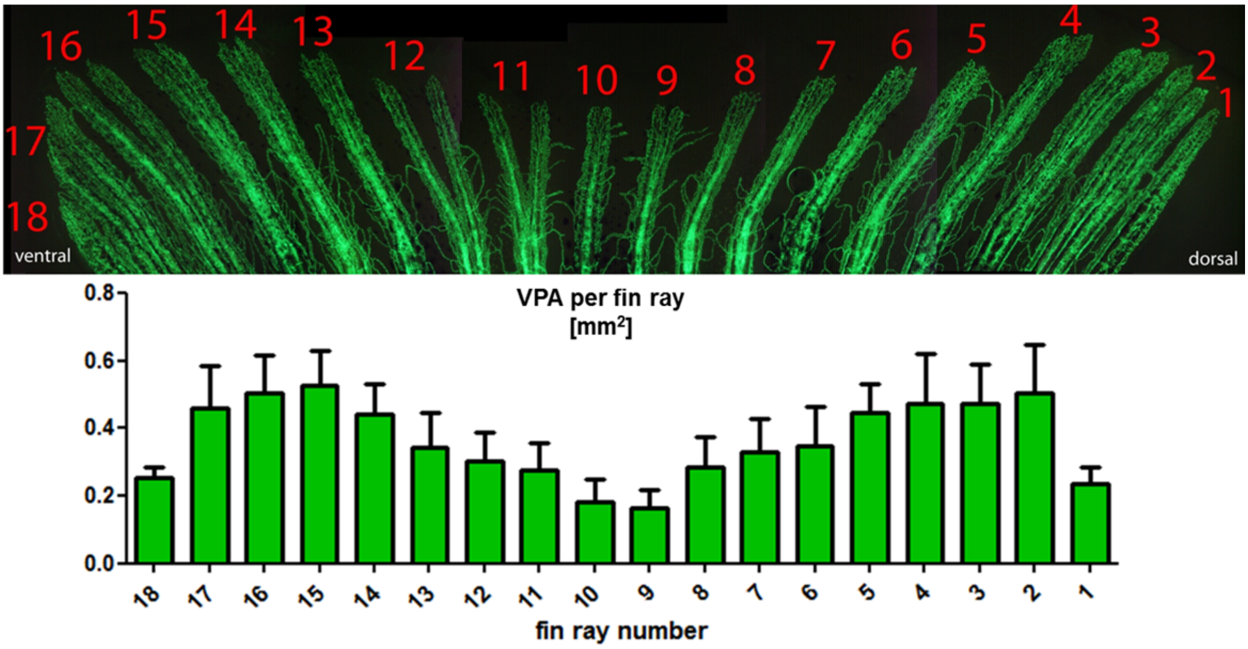

S4 Figure. *Identification and quantification of individual fin rays (n = 7)*

Stereology allowed quantification of absolute values for various parameters from individual fin rays. This can be very important to reduce the area of interest to specific sites. In this example, the fin rays were numbered from dorsal to the ventral aspect.
