# Supplementary material for: Zebrafish Caudal Fin Angiogenesis Assay—Advanced Quantitative Assessment Including 3-Way Correlative Microscopy
Source: PLoS One. 2016 Mar 7;11(3):e0149281. doi: 10.1371/journal.pone.0149281 (PMC4780710; doi:10.1371/journal.pone.0149281)
Supplement: S5 Fig — (PDF) [file pone.0149281.s005.pdf]

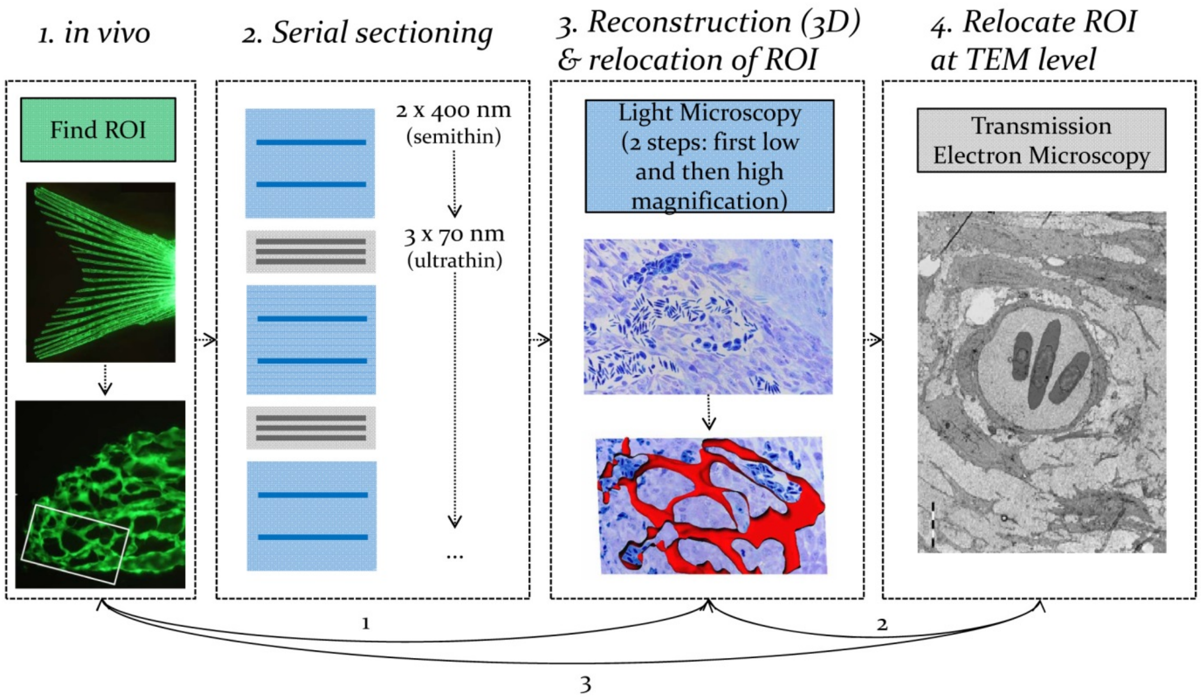

S5 Figure. *Scheme and detailed description of the 3-way correlative microscopy.*

1. After defining the region of interest (ROI) during the in vivo imaging the fin ray of interest with the 2 neighboring fin rays (on each side – altogether 5 fin rays) were harvested. 5 rays were taken for easier orientation afterwards – the fin ray of interest was always in the middle.

2. After harvesting the sample was put into a special container consisting of two flat metallic nets closely pressed to one another for further proceeding. It was necessary for the fin to stay as flat as possible – otherwise it easily deforms during the fixation and subsequent dehydration steps. Finally, the sample was embedded and cut longitudinally: 2 semithin sections (400nm-thick) were followed by 3 ultrathin sections (70 nm-thick). The whole fin ray was cut through in this way.

3. Step 1 (optional): From the obtained serial semithin sections the images at x20 magnification were taken, aligned using Adobe Photoshop 6.0 and the vessel were marked with red color in a user-driven way. The obtained 3D-stack was uploaded and analysed using Imaris Software: from the obtained reconstruction of the vasculature one could orient himself and relocate region of interest in the semithin sections.

An experienced user would not need to have the full 3D reconstruction to orient himself on the semithin section – so this step can actually be omitted.

Step 2. To precisely localize the ROI and to be able to compare high magnification in vivo images with the findings in semithin sections a 3D reconstruction based on the x63 images from serial semithin sections was done. This step is needed to drastically improve the efficiency of the correlative TEM (the search is limited to a defined area in only 2-3 serial ultrathin sections).
