# Supplementary material for: Zebrafish Caudal Fin Angiogenesis Assay—Advanced Quantitative Assessment Including 3-Way Correlative Microscopy
Source: PLoS One. 2016 Mar 7;11(3):e0149281. doi: 10.1371/journal.pone.0149281 (PMC4780710; doi:10.1371/journal.pone.0149281)
Supplement: S1 Table — (PDF) [file pone.0149281.s006.pdf]

**S1 Table.** *Formulary*

| Full name | Abbrev. | Unit | Formula | Explanation |
|---|---|---|---|---|
| Area (stereology) | TRA, VPA | [$mm^2$] | $\mathrm{Area} = N_p * \left(\frac{\mathrm{area}}{\mathrm{point}}\right)$ | *Where* $N_p$ *is the number of points obtained by counting, area/point= the applied size of the grid (point associated area) dL= distance between grid lines.* |
| Length (stereology) | CL | [m] | $Length = N_p * \frac{\pi}{2} * dL$ | |
| Vessel Area Density | VAD | [%] | $\mathrm{VAD} = \frac{\mathrm{VPA}}{\mathrm{TRA}} * 100$ | |
| Average Vessel Diameter | D | [µm] | $\mathrm{d} = \frac{2 * \mathrm{VPA}}{\mathrm{CL}}$ | *Assumptions that vessels are round structures and the diameter of a vessel is smaller than its length.* |
| Vascular Exchange Surface | VES | [$\mu m^2$] | $\mathrm{VES} = \pi * \mathrm{VPA}$ | *We assessed the vascular exchange surface, as the area of the outer surface of the vasculature. Assumption that vessels are cylindric structures.* |
