# Supplementary material for: Zebrafish Caudal Fin Angiogenesis Assay—Advanced Quantitative Assessment Including 3-Way Correlative Microscopy
Source: PLoS One. 2016 Mar 7;11(3):e0149281. doi: 10.1371/journal.pone.0149281 (PMC4780710; doi:10.1371/journal.pone.0149281)
Supplement: S1 Text — (PDF) [file pone.0149281.s007.pdf]

S Text. Info on the graph energy

From the skeleton-derived graph (branching points=nodes), the adjacency matrix A can be constructed. It consists in a double array A = [aij], whose entries aij are the number of edges connecting node *i* to node *j.* There are several properties related with enumerating walks and connectivity for the adjacency matrix that have received an increased attention in the context of urban transport problems, social networks analysis (i.e. the study of human interactions with graph theoretical tools)[1] etc. The spectrum of a graph, defined as the set of eigenvalues derived from the adjacency matrix also shed light about the structure of the graph (subject of Spectral Graph Theory). The energy of the graph, defined to be the sum of the absolute values of the eigenvalues of the adjacency matrix of that graph, rose from theoretical chemistry where eigenvalues were associated with the stability of molecules[2]. To mention some properties of the eigenvalues of the graph, the eigenvalues are arranged in a decreasing sequence. The Perron–Frobenius Theorem implies immediately that if the graph is connected, then the largest eigenvalue has multiplicity 1 and this eigenvalue is an *average* degree (i.e. number of incident edges to a node) for the graph[3]. Non-connected nodes or components translate in zero entries in the adjacency matrix providing zero-valued eigenvalues. Due to the non-negative nature of the absolute value function, any added node will either have any impact on the energy of the graph (i.e. the associated eigenvalue will be zero in case of isolated nodes) or a magnitude proportional to its degree (i.e. eigenvalue will have a non-negative contribution to the graph energy being larger if the node is more connected). Therefore, the larger the graph energy, the more connected is the graph and consequently this quantity expresses the connectivity between the nodes.

1) Skillicorn, D. B. (2005) Social Network Analysis via Matrix Decompositions, in Emergent Information Technologies and Enabling Policies for Counter-Terrorism (eds R. L. Popp and J. Yen), John Wiley & Sons, Inc., Hoboken, NJ, USA. Chapter 19.
2) Gutman, Ivan (1978), "The energy of a graph", 10. Steiermärkisches Mathematisches Symposium (Stift Rein, Graz, 1978), Ber. Math.-Statist. Sekt. Forsch. Graz 103, pp. 1–2
3) Kay Buttler, Steven (2008) Eigenvalues and Structures of Graphs. PhD dissertation, University of California, San Diego.
